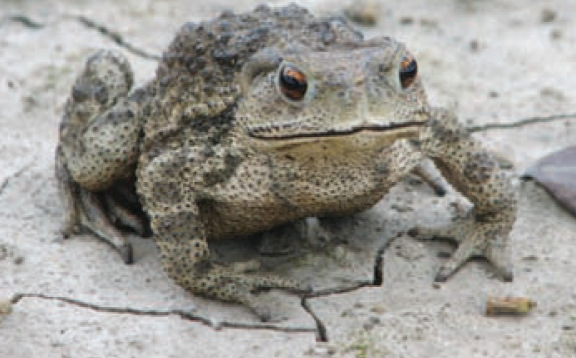# The Beat

**Published:** 2008-03

## Savory Relief for Arsenic Poisoning?

In the February 2008 issue of *Food and Chemical Toxicology*, researchers from India report that aqueous garlic extract (AGE) fed to at-risk individuals may reduce the toxic effects of arsenic. Rats receiving daily doses of arsenic equivalent to the levels in groundwater from heavily arsenic-contaminated areas of the Bengal Basin retained significantly less of the element in blood and liver and excreted significantly more in urine when fed 2 mg/mL AGE. The researchers believe the antioxidant properties of garlic, along with the chelating efficacy exhibited, led to the success of the treatment. AGE was also seen to significantly reduce intracellular reactive oxygen species in several cell types.

**Figure:**
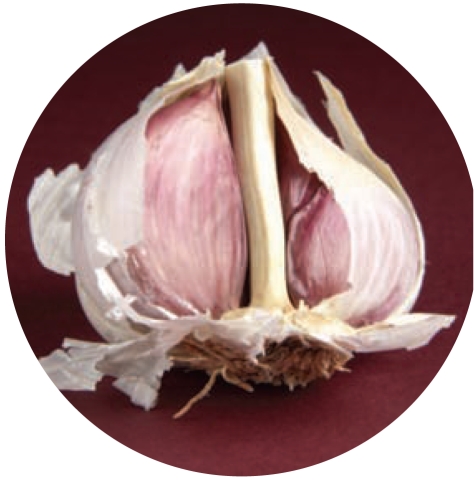


## *In Utero* Cigarette Smoke Exposure and Age at Menopause

It is well documented that women who smoke cigarettes begin menopause earlier than nonsmokers. Using data from a U.S. national study on the health effects of prenatal diethylstilbestrol exposure, a team of researchers reported in the 11 January 2008 advance access edition of the *American Journal of Epidemiology* that study participants who had never smoked cigarettes but had been prenatally exposed to maternal cigarette smoke experienced earlier-onset menopause. Moreover, previously noted associations between current smoking and age at menopause were not observed among these women.

## Antibiotic Resistance Seen in Arctic Wildfowl

Swedish researchers reported in the January 2008 issue of *Emerging Infectious Diseases* that birds living in three different geographic regions of the Arctic tundra carry *E. coli* bacteria resistant to multiple types of antibiotics. These birds, which lived in northeastern Siberia, northern Alaska, and northern Greenland, are believed to have had no contact with humans. The researchers proposed three possible explanations for their findings: the birds could have been exposed to the bacteria through contact with other species of birds migrating from other regions, or resistance could have developed either through spontaneous mutations or through horizontal gene transfer from other microbes.

**Figure:**
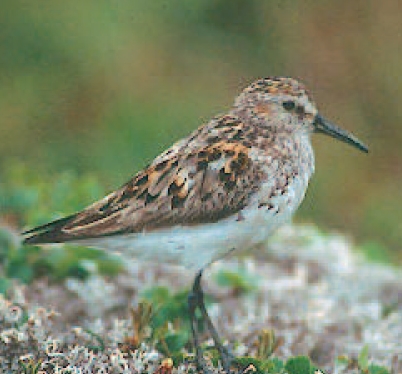
The western sandpiper, one of the Arctic species studied

## UK Organic Certifier Says No to Nanomaterials

The Soil Association, a nonprofit UK organization that certifies the majority of that nation’s organic products, announced in January 2008 it will no longer allow man-made nanomaterials (materials with a mean size of less than 200 nm) to be used in goods carrying its certification label. This rule, the first of its kind in the world, will apply mainly to personal care products but also could apply to other categories such as food and textiles. The group cites insufficient evidence on the impact of nanomaterials on the environment and human health.

**Figure:**
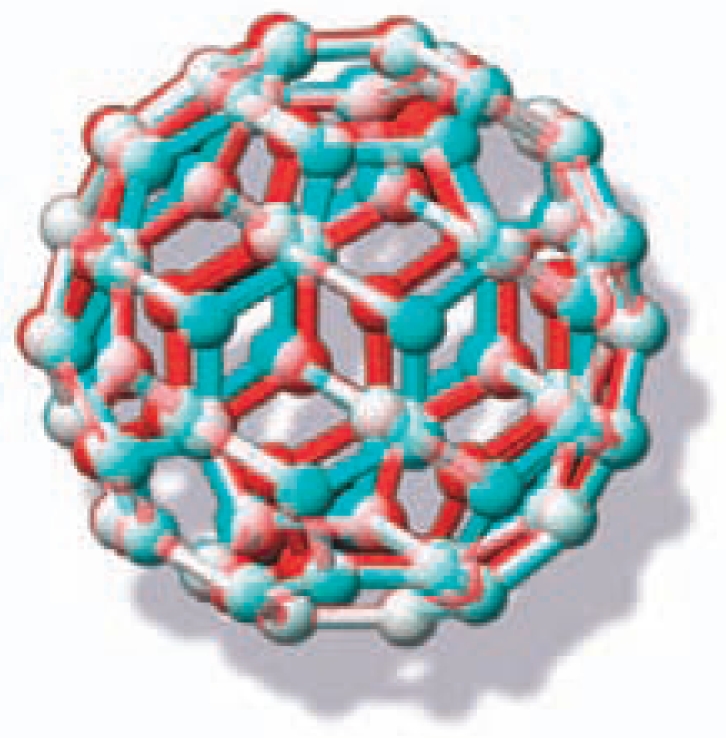


## China Begins National Survey of Pollution Sources

Although China has set goals to cut emissions of major pollutants, emissions of sulfur dioxide and some indicators of poor water quality increased in 2006. Experts have decried the lack of trustworthy statistics on the sources and extent of pollution and the number of remediation facilities. In response, the Chinese government invested US$100 million in 2007 to launch the collection of data from industrial, agricultural, and residential sources at sites throughout China, beginning in February 2008. The work, which is being overseen by the State Environmental Protection Administration and the Ministry of Agriculture, will also compile information on methods of environmental remediation currently available in the country. Data collection is projected to be complete by mid-2008.

## Ancient Chinese Cancer Secret?

Researchers at the University of Texas M.D. Anderson Cancer Center are currently conducting clinical trials in Shanghai on a cancer therapy that uses the venom of the Asiatic toad (*Bufo gargarizans*). China’s use of the venom in the treatment of a number of illnesses can be traced back to the tenth century. In the clinical trials, compounds from the venom have proven beneficial and caused no apparent side effects in patients with advanced liver, pancreatic, or lung cancer. In mouse studies, the compound was better at shrinking pancreatic tumors than gemcitabine, a standard chemotherapy drug. Cardiac glycosides in the venom are thought to inhibit proteins that promote cancer cell growth, thereby causing cancer cell death. Although potentially toxic at high doses, these glycosides are used to treat congestive heart failure. Currently, the venom-based treatment is administered by injection, but the Texas researchers hope to develop a pill form.

**Figure:**